# Multiple *Francisella tularensis* Subspecies and Clades, Tularemia Outbreak, Utah

**DOI:** 10.3201/eid1412.080482

**Published:** 2008-12

**Authors:** Jeannine M. Petersen, Jennifer K. Carlson, Gabrielle Dietrich, Rebecca J. Eisen, Jana Coombs, Aimee M. Janusz, JoDee Summers, C. Ben Beard, Paul S. Mead

**Affiliations:** Centers for Disease Control and Prevention, Fort Collins, Colorado, USA (J.M. Petersen, J.K. Carlson, G. Dietrich, R.J. Eisen, A.M. Janusz, C.B. Beard, P.S. Mead); Utah Department of Health, Salt Lake City, Utah, USA (J. Coombs, J. Summers)

**Keywords:** Tularemia, Francisella tularensis, tick-borne diseases, Utah, dispatch

## Abstract

In July 2007, a deer fly–associated outbreak of tularemia occurred in Utah. Human infections were caused by 2 clades (A1 and A2) of *Francisella tularensis* subsp. *tularensis*. Lagomorph carcasses from the area yielded evidence of infection with A1 and A2, as well as *F. tularensis* subsp. *holarctica*. These findings indicate that multiple subspecies and clades can cause disease in a localized outbreak of tularemia.

Tularemia is a zoonotic disease caused by *Francisella tularensis,* a highly infectious, gram-negative coccobacillus found in lagomorphs (rabbits and hares), rodents, and arthropods throughout the Northern Hemisphere. Humans become infected through contact with infected animal tissues, ingestion of contaminated food or water, inhalation of contaminated aerosols, and bites of arthropods, especially ticks and deer flies.

In North America, tularemia is caused by 2 subspecies of *F. tularensis*, subsp. *tularensis* (type A) and subsp. *holarctica* (type B). The distribution of type A and type B strains appears largely overlapping within the United States, with some geographic distinctions (*1*,*2*). Ecologically, the 2 subspecies are thought to be maintained in distinct but incompletely defined cycles, with type A strains frequently associated with lagomorphs and type B strains more commonly associated with rodents and aquatic environments (*3*).

Type A strains can be further divided into 2 major clades by various molecular subtyping techniques (*1*,*2*,*4*–*7*). These clades, designated here as A1 and A2, differ in their overall geographic distribution and clinical features. A1 strains (also known as A.I. and A-east) are usually found east of the Rocky Mountains. A2 strains (also known as A.II. and A-west) are common in the intermountain region of the western United States, are associated with lower mortality rates in humans, and are the only strains currently linked to transmission by deer flies (*Chrysops* spp.) (*2*).

## The Outbreak

**Figure 1:**
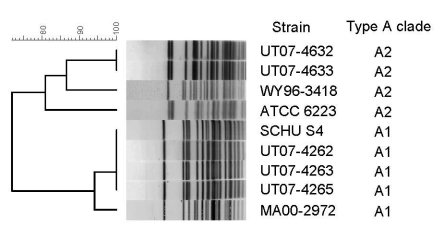
Dendrogram based on *Pme*I pulsed-field gel electrophoresis (PFGE) patterns of *Francisella tularensis* type A isolates. The dendrogram was constructed by using Dice similarity coefficients (1.5% optimization and 1.5% tolerance) and unweighted pair group method with averages. Strains WY96–3418, ATCC 6223, SCHU S4, and MA00–2972 were included as known A1 and A2 controls for creation of the dendrogram. Control strains were previously identified as either A1 or A2 by multiple methods including multilocus variable number tandem repeat analysis, PFGE, housekeeping gene sequence analysis, whole genome sequencing, and Indel analysis (*1*,*2*,*4*–*7*,*9**).*

In July 2007, an outbreak of ulceroglandular tularemia occurred in Utah among visitors to the southwest shore of Utah Lake; an epidemiologic investigation implicated deer fly bites as the source of infection (*8*). Clinical isolates were obtained July 9–14 from skin lesions of 5 patients. Isolates were identified as *F. tularensis* subsp. *tularensis* (type A) by biochemical analysis (glycerol fermentation). Molecular subtyping of isolates was performed by using *Pme*I pulsed-field gel electrophoresis (PFGE) as previously described (*2*). PFGE gels were normalized by comparison to the *Salmonella enterica* serotype Braenderup (H9812) reference strain by using BioNumerics software (v. 4.0, Applied Maths BVBA, Sint-Martens-Latem, Belgium) (*2*). A dendrogram was constructed by comparison with *Pme*I PFGE patterns for A1 (SCHU S4, MA00–2972) and A2 (ATCC 6223, WY96–3418) control strains (*1*,*2*,*4*–*7*,*9*) (Figure 1). The 5 clinical isolates fell into the 2 major type A clades; 2 isolates were identified as A2 (UT07–4632, UT07–4633), and 3 were identified as A1 (UT07–4262, UT07–4263, UT07–4265) (Figure 1). PFGE patterns for the 3 A1 isolates were indistinguishable from each other, as were patterns for the 2 A2 isolates (Figure 1).

**Figure 2:**
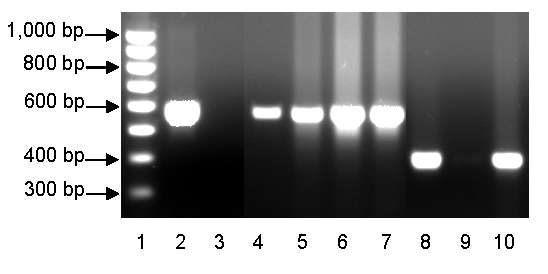
PCR typing of *Francisella tularensis*, clades A1 and A2, in dessicated lagomorph carcasses. Lane 1, 100-bp ladder (Bio-Rad, Hercules, CA, USA); lane 2, A1 positive control (Schu S4); lane 3, A1 negative control (NM99); lane 4, UT07–5156 (A1); lane 5, UT07–5152 (A1); lane 6, UT07–5157 (A1), lane 7, UT07–5159 (A1), lane 8, A2 positive control (NM99); lane 9, A2 negative control (Schu S4); lane 10, UT07–5161 (A2).

A brief search of the exposure area yielded desiccated carcasses of 10 black-tailed jackrabbits (*Lepus californicus)* and 2 desert cottontail rabbits (*Sylvilagus audubonii*). The carcasses were found within a few hundred meters of each other, in an overall area <0.8 km across. Living deer flies (*Chrysops* spp.) were collected from the same area. DNA was extracted from lagomorph bone marrow and flies by using the QIAamp DNA MiniKit (QIAGEN, Valencia, CA, USA) and tested with real-time PCR *F. tularensis* multitarget, type A and type B assays (*10*,*11*). Although all deer fly samples were negative, 11 of 12 lagomorph carcasses tested positive for *F. tularensis* by the multitarget assay (3 of 3 targets positive; crossing threshold (C_t_) range 13–38). Among the infected samples, 9 tested positive for type A (C_t_ range 13–36) and 2 tested positive for type B (C_t_ range 19–24). The subtyping results were verified by sequencing of the succinate dehydrogenase gene (*sdhA*), which distinguishes type A and type B strains on the basis of a single nucleotide polymorphism (*12*). Type B strains have a G at nt 465 of the *sdhA* gene sequence, whereas type A strains have an A at this position. To further distinguish the type A samples between clade A1 or clade A2, conventional PCRs were used (*13*). Sufficient *F. tularensis* DNA was present to type infections for 5 of the 9 type A–positive lagomorph carcasses; 4 yielded a PCR product consistent with the A1 clade (570 bp), and 1 yielded a product consistent with the A2 clade (396 bp) (Figure 2) (*13*).

## Conclusions

Few studies have reported on the diversity of *F. tularensis* subsp. or clades present during outbreaks of tularemia, in part because molecular methods for strain discrimination have only recently been described (*1*,*2*,*4*–*7*). In this discrete deer fly–associated outbreak, we found human infections caused by both A1 and A2 strains, and evidence that A1, A2, and type B strains were circulating among lagomorphs in the exposure area. These findings demonstrate that multiple subspecies and clades can cause disease in a localized outbreak of tularemia and that deer-flies are associated with transmission of A1 strains.

Published reports indicate that A1 and A2 strains are generally segregated into areas east and west of the Rocky Mountains, respectively, with some overlap in coastal California (*1*,*2*). In contrast, our results demonstrate that A1 strains are present in areas of the intermountain west and at elevations >1,200 m. This finding is supported by identification of an additional case of human tularemia in Utah caused by an A1 strain in 1998 (Centers for Disease Control and Prevention, unpub. data). On a local level, our results indicate that A1, A2, and type B strains can coexist naturally within the same ecosystem, a paradox when compared with the segregation that appears to exist on a larger scale. Overall, these observations underscore the need for future studies to define the ecologic and evolutionary factors underlying the distributions of *F. tularensis* strains in North America.

Although the role of deer flies as vectors of *F. tularensis* is well established, the dynamics of deer fly–associated outbreaks have not been well researched. Transmission of *F. tularensis* by deer flies is believed to be entirely mechanical, through contamination of the mouthparts. Long-term maintenance of *F. tularensis* has not been shown to occur in deer flies, and it is therefore not surprising that the deer flies we collected 3 weeks after the outbreak tested negative for this organism. Our findings suggest that deer flies nonselectively acquire and transmit whatever strains are circulating in enzootic hosts. We postulate that, in this instance, an abundance of deer flies led to extensive feeding on many hosts, resulting in the simultaneous transmission of multiple strains. High mortality rates among lagomorphs may have forced deer flies to seek alternate hosts, specifically muskrats, which are associated with type B strains and have been linked to outbreaks among trappers at Utah Lake (*3*).

The co-occurrence of multiple subspecies and clades may be unique to arthropod-associated outbreaks of tularemia and not characteristic of outbreaks resulting from other modes of *F. tularensis* transmission, such as contaminated water. Further work is needed to determine whether our findings will apply to other deer fly–associated outbreaks or for outbreaks of tularemia associated with ticks, which are known to maintain as well as transmit *F. tularensis*. Notably, while investigating a tick-borne outbreak of presumed type B infections in South Dakota, Markowitz and colleagues found evidence of both type A and type B strains in *Dermacentor variabilis* ticks collected from dogs (*14*). Outbreaks involving multiple serotypes have been observed with other vector-borne pathogens, including dengue virus (*15*), which suggests that amplification and transmission of multiple strains in a focal area may represent a general feature of some vector-borne diseases.
